# DYRK1A antagonists rescue degeneration and behavioural deficits of in vivo models based on amyloid-β, Tau and DYRK1A neurotoxicity

**DOI:** 10.1038/s41598-022-19967-y

**Published:** 2022-09-23

**Authors:** Bangfu Zhu, Tom Parsons, Christopher Foley, Yeng Shaw, Travis Dunckley, Christopher Hulme, James J. L. Hodge

**Affiliations:** 1 School of Physiology, Pharmacology and Neuroscience, Faculty of Life Science, University of Bristol, Biomedical Sciences Building, University Walk, Bristol, BS8 1TD UK; 2 Department of Chemistry and Biochemistry, University of Arizona, Tucson, USA; 3 Division of Drug Discovery and Development, Department of Pharmacology and Toxicology, College of Pharmacy, The University of Arizona, Tucson, AZ 85721 USA; 4 Neurodegenerative Disease Research Center, Biodesign Institute, Arizona State University, Tempe, AZ 85281 USA

**Keywords:** Alzheimer's disease, Neurodegeneration, Circadian rhythms and sleep, Learning and memory, Motor control, Neural ageing, Olfactory system, Phenotypic screening

## Abstract

Alzheimer’s disease (AD) involves pathological processing of *amyloid precursor protein* (*APP*) into amyloid-β and *microtubule associated protein Tau* (*MAPT)* into hyperphosphorylated Tau tangles leading to neurodegeneration. Only 5% of AD cases are familial making it difficult to predict who will develop the disease thereby hindering our ability to treat the causes of the disease. A large population who almost certainly will, are those with Down syndrome (DS), who have a 90% lifetime incidence of AD. DS is caused by trisomy of chromosome 21 resulting in three copies of *APP* and other AD-associated genes, like dual specificity tyrosine-phosphorylation-regulated kinase 1A (*DYRK1A*) overexpression. This implies that DYRK1a inhibitors may have therapeutic potential for DS and AD, however It is not clear how overexpression of each of these genes contributes to the pathology of each disease as well as how effective a DYRK1A inhibitor would be at suppressing any of these. To address this knowledge gap, we used *Drosophila* models with human *Tau*, human *amyloid-β* or fly *DYRK1A* (*minibrain* (*mnb*)) neuronal overexpression resulting in photoreceptor neuron degeneration, premature death, decreased locomotion, sleep and memory loss. DYRK1A small molecule Type 1 kinase inhibitors (DYR219 and DYR533) were effective at suppressing these disease relevant phenotypes confirming their therapeutic potential.

## Introduction

AD is the most prevalent form of dementia with 42.3 million people with AD worldwide in 2020. With ageing populations, this number set to increase further, along with the economic costs that are already estimated to be $818 billion^1^. There remains no cure or treatments for the causes of AD, with only a limited number of drugs that treat some symptoms in some people. Cholinergic neuron loss in the hippocampus and cortex is prominent, leading to compromised memory, with extensive neurodegeneration finally leading to premature death^2,3^. AD is also associated with disrupted circadian rhythms and sleep^4–8^. Post-mortem slices allow staining of extracellular plaques of amyloid-β (Aβ) consisting of neurotoxic Aβ peptides such as the 42 amino acid (aa) peptide (Aβ42). Aβ42 is the product of amyloidogenic cleavage of amyloid precursor protein (APP) by secretases. Mutations in APP and within some of these secretases are a cause of familial AD^9^. The synapse loss and cognitive decline evident later in AD most strongly correlate with the accumulation of intracellular tangles of hyperphosphorylated Tau protein. Tau is phosphorylated by multiple kinases, including GSK-3β, CDK5, JNK, MAPK, CaMKII and DYRK1A^10,11^. Tau is the product of the microtubule associated protein tau (MAPT) gene, which exists as six isoforms with variable numbers of N-terminal domains (e.g. 0 N, 1 N or 2 N) and C-terminal tubulin binding repeats (e.g. 3R or 4R). Therefore, the reason why 4R Tau is more strongly associated with AD is that it contains four self-aggregating repeats compared to three in the 3R isoform making it more prone to aggregate and cause AD pathology^12,13^.

About 5% of AD are early-onset familial forms due to autosomal dominant inheritance of *APP, PSEN1* and *PSEN2* causative mutations, with *APOE*ε4 allele being the main genetic risk factor for sporadic AD^14^. This has also obstructed the prediction of who and when people will get AD, with diagnosis often only being confirmed post-mortem or after irretrievable loss of neurons, hence frustrating attempts at early intervention and development of causal treatments^15,16^. Most of our understanding of the mechanisms that underly AD pathology are limited to rodent models of familial AD based on misexpression of different APP or Tau transgenes that do not recapitulate progressive neurodegeneration and all of the behavioral hall marks of the disease. These genetic models may not fully recapitulate ~ 95% of the causes of AD, which are sporadic. This could partially explain the lack of translation of drugs that are effective in these models to clinical efficacy^2,15–18^. Therefore, there is a need for more diverse animal models that faithfully represent the pathology of both familial and sporadic causes of the disease and that allow high-throughput screening in a genetically and experimentally tractable system.

*Drosophila*, holds some potential in this regard with neuronal overexpression of different human APP and Tau products leading to photoreceptor neuron degeneration, shortened lifespan and deficits in neuronal excitability, movement, circadian rhythm, sleep and memory^18–28^.

One large (1:450–1:2200 live births) cohort of people that can be predicted to develop AD (90% lifetime incidence) and which can be diagnosed in utero and then followed from birth are those with Down syndrome (DS) or trisomy 21. DS is caused by expression of three copies of chromosome 21 genes, including overexpression of APP and hence increased AD pathology^2,29^. DS is a syndrome involving complex developmental abnormalities leading to compromised motor control, learning difficulties and sleep disruption^30–32^. People with DS develop Alzheimer’s disease (AD)-like (AD-DS) pathology of Aβ plaques and accumulation of tangles of hyperphosphorylated Tau leading to neurodegeneration in people as young as 20 years old, causing progressive motor, cognitive and health decline in early middle age^33,34^ and, finally, shortening of life by ~ 30 years^35^. Therefore, overexpression of chromosome 21 genes may not only cause DS but also be an early driver of AD pathology. One such candidate gene is *DYRK1A,* which is highly expressed throughout the brain and further so in DS^36,37^, contributing to motor and cognitive deficits associated with the disease^11,29,33,38–40^. DYRK1A phosphorylates Tau at S396, T231, and S262 as well as changes the splicing of Tau isoforms, resulting in increased neurofibrillary tangle formation^41–47^. DYRK1A phosphorylates APP and promotes neurotoxic Aβ peptide processing^11,38,39^. Thus, DYRK1A impacts both major pathology pathways of AD.

The fly ortholog of *DYRK1A* is *mnb*, that exists in five isoforms, *E-I*, which all contain the highly conserved kinase domain^48^. Mnb is a presynaptic protein, mutations in which disrupt growth of presynaptic boutons and recycling of transmitter vesicles. Expression of *mnb*-*F* rescues these defects^49^. Neuronal expression of three copies of the longest isoform *mnb-H* (1.5 × wildtype levels) increased the number of glutamatergic boutons, enhanced spontaneous vesicular transmitter release, and slowed recovery from short-term depression resulting in motor impairment that persisted in aged animals. These animals also displayed progressive neurodegeneration and shortened lifespan^50^.

Like AD there are no treatments for DS, with a lack of models and knowledge of underlying mechanisms hampering development of new drug targets and treatments. However, kinase inhibitors of DYRK1A kinase have shown efficacy in preclinical models of DS and AD^37,38,45^. The DYRK1A benzimidazole inhibitor DYR219 decreased Aβ42 aggregation and Tau phosphorylation, reversing cognitive deficits in 3xTg-AD mice that overexpress mutant APP K670M/N671L, MAPT P301L and Presenilin M146V^46,51^. The DYRK1A inhibitor SM07883 also reduced Tau hyperphosphorylation in mice overexpressing MAPT P301L, leading to decreased Tau aggregation, neurodegeneration and improvements in behavioural deficits^44^. Leucettine inhibitor (L41) treatment of DYRK1A overexpressing mice ameliorated brain connectivity and synaptic protein expression, rescuing cognitive deficits^52^. The drug CX-4945 is a competitive inhibitor of the ATP-binding site of DYRK1A which is required for its kinase activity, hence was able to correct the hyperphosphorylation of Tau, APP and PS1 caused by DYRK1A overexpression in cell lines^47^. In *Drosophila* CX-4945 decreased fly eye degeneration caused by human Tau overexpression and delayed lethality resulting from pan-neuronal overexpression of mnb-H^47^. Likewise, in mice overexpressing DYRK1A, CX-4945 decreased Tau hyperphosphorylation^47^. In addition, some of these drugs may also induce protein degradation of DYRK1A or movement of APP to lysosomes for degradation, thereby providing additional means to correct DS and AD pathology^45,51^. Therefore, DYRK1A inhibitors hold great promise for the treatment of DS and AD pathology. To characterise the novel DYRK1A inhibitors, DYR219 and DYR533, we compared the effects of overexpression of human Tau (0N4R), human tandem oligomerising secreted Aβ42 and fly mnb-H on degeneration of photoreceptor neurons, longevity, motor performance, sleep and memory measuring the ability of the drugs to suppress these DS and AD relevant phenotypes.

## Results

### DYRK1A kinase inhibitors block mnb phosphorylation of human Tau expressed in *Drosophila* neurons

**Figure 1:**
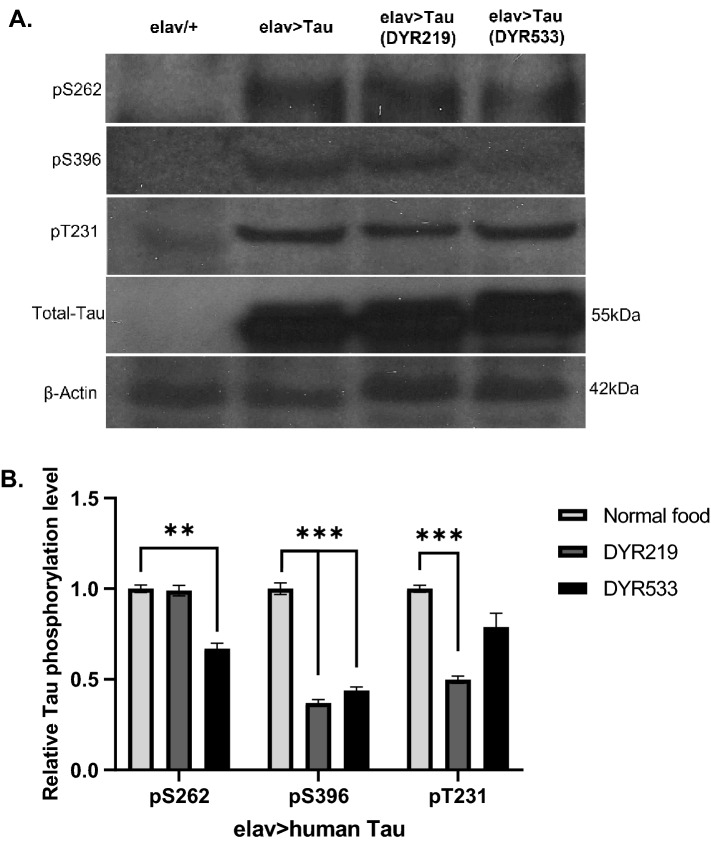
DYRK1A inhibitors reduced phosphorylation of human Tau expressed pan-neuronally in *Drosophila*. Western blots show effects of treatment with DYRK1A inhibitors (DYR219 and DYR533) on phosphorylation of human Tau 0N4R overexpressed pan-neuronally using the *elav-Gal4* driver: 1st lane is *Elav-Gal4/* + control, 2nd lane is *Elav* > *human Tau 0N4R* with normal food, 3rd lane is *Elav* > *human Tau* treated with 304 μM DYR219 and 4th lane is *Elav* > *human Tau* with 248 μM DYR533 treatment throughout development and adulthood (treatments were the same for all subsequent figures). Three antibodies against phosphorylated Tau (pS262, pS396 and pT231) were tested compared to the level of total unphosphorylated Tau (~ 55 kDa) using a fourth antibody. At the bottom is the β-actin protein loading control (~ 42 kDa). Supplemental Fig. S1 shows the full Westerns that the bands were imaged from. Intensity of bands are presented as mean ± Standard Error of the Mean (SEM) with data compared by 2-way ANOVA with Dunnett's multiple comparisons test **P < 0.01 and ***P < 0.001.

The generic structures of DYR219 which has a K_D_ for DYRK1A of 16 nM and DYR533 which has a K_D_ for DYRK1A of 1.4 nM have been described in two separate patents^53,54^. Based on the food concentration of DYR219^54^ that was previously shown to decrease Aβ42 and Tau pathology and to reverse cognitive deficits of AD mice, we fed control (*elav-Gal4/*+) or flies overexpressing AD associated human Tau 0N4R in all neurons (*elav* > *Tau*) 304 μM DYR219 and 248 μM DYR533. The *UAS-human MAPT (TAU 0N4R)*^28^, has been reported to be leaky^55^, however we found *UAS-Tau 0N4R/* + controls were wildtype for many of the phenotypes reported here^56^, consistent with this not being a concern*.* Feeding these concentrations of drugs throughout development and adulthood produced viable offspring. Total protein was extracted from whole heads from control and human Tau expressing flies treated with the different drugs or vehicle and was equal in quantity in all lanes (Fig. 1A with the full version of Western blots shown in Supplemental Fig. S1). We selected widely used phospho-specific antibodies to the main sites on human Tau (e.g. pT231, pS262 and pS396) directly phosphorylated by DYRK1A^36,43–47^. The Western blots showed that the levels of phosphorylated Tau were reduced at sites pT231 and pS396 with DYR219 treatment and reduced at sites pT231, pS262 and pS396 with DYR533 treatment compared to untreated Tau overexpressors. *elav* > *Tau* was found to cause a robust overexpression of human Tau detected using an antibody to full length human Tau, which only reported a small amount of endogenous fly Tau (visible in *elav/* + lane). DYR treatment did not cause a visible change in the level of total unphosphorylated Tau. Quantification of intensity of bands from three Western blots showed the levels of phosphorylated Tau were indeed reduced at sites pT231 and pS396 with DYR219 treatment (Fig. 1B). DYR533 significantly reduced phosphorylation of Tau at S262 and S396, with the reduction in pT231 intensity not being significantly reduced. As these concentrations of drug and treatment protocol was effective at suppressing pathological phosphorylation of Tau in vivo, the same drug treatment protocol was used in all subsequent experiments.

### Pharmacological inhibition of DYRK1A decreases AD and DS degenerative phenotypes

**Figure 2:**
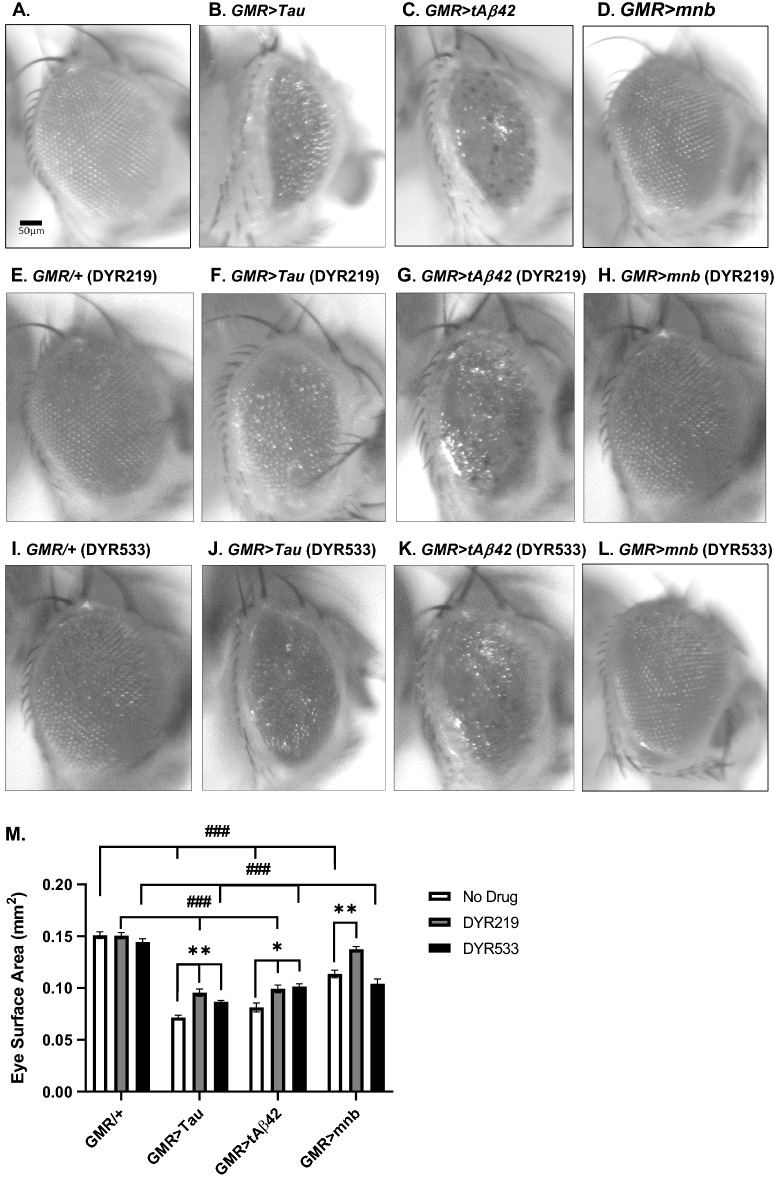
Overexpression of human Tau, amyloid-β42 and mnb in the eye caused degeneration of the photoreceptor neurons suppressed by DYRK1A inhibitors. (**A**) (*Left*) Images of adult fly eyes of control (*GMR-Gal4/* + with scale bar 50 μm) and those overexpressing human Tau 0N4R isoform (**B**), human secreted tandem oligomerising amyloid-β42 (**C**) or (minibrain) mnb isoform H (**D**) throughout development and adulthood. Note the prominent rough eye phenotypes and reduced eye size in flies overexpressing the neurotoxic genes. DYRK1A inhibitors did not affect the eye size of controls (*GMR-Gal4/* +) fed DYR219 (**E**) or DYR533 (**I**). However, DYR219 suppressed the reduction in eye size and rough eye phenotype of the degenerative mutants overexpressing Tau (**F**), tAβ42 (**G**), and mnb (H). Likewise, DYR533 was effective at treating degenerative phenotypes caused by Tau (J), tAβ42 (**K**) but not mnb (**L**). (**M**) Eye degeneration was quantified by eye surface area (n = 7 per genotype and drug treatment group) with flies overexpressing human Tau, human Aβ42 and mnb having smaller eyes than control (^###^*P* < 0.001, 2-way ANOVA with Dunnett's post hoc multiple comparisons test). DYR219 increased the size of these smaller eyes (*P < 0.05 and **P < 0.01), including completely rescuing the size of the mnb overexpressing eye to a level indistinguishable from control. DYR533 increased the size of the degenerative mutants except mnb.

As human Tau, amyloid-β and DYRK1A have been associated with neurodegeneration in AD and AD-DS, we wished to confirm if these molecules were also neurotoxic in *Drosophila.* We overexpressed in the eye throughout developmental and adulthood human Tau 0N4R, human amyloid-β42 (Aβ42) and the fly orthologue of DYRK1A called *minibrain* (*mnb*) using the *Glass multimer reporter* (*GMR-Gal4*) promoter^24,50^. Compared to control (*GMR-Gal4/* + *,* Fig. 2A), which have large and regularly aligned ommatidia of their compound eye, overexpression of the disease associated genes caused neurotoxicity resulting in loss of photoreceptors neurons and a “rough eye” phenotype due to misalignment and disorganisation of the compound eye (Fig. 2B–D). To quantify this phenotype, we measured the surface area of the eye overexpressing human Tau (~ 60% reduction in area), human Aβ42 (~ 50% reduction) and mnb (~ 30% reduction) whose loss of photoreceptor neurons resulted in a decrease in eye size compared to control (Fig. 2M). DYR219 and DYR533 treatment did not affect the appearance of control eyes (Fig. 2E,I) or their size (Fig. 2M). However, DYR219 was able to suppress the rough eye and smaller eye phenotype of Tau (Fig. 2F), Aβ42 (Fig. 2G) and mnb (Fig. 2H) causing a significant increase (~ 10–20% increase in area) in each genotype’s eye size (Fig. 2M). Therefore, partially rescuing Tau and Aβ42 degeneration while completely rescuing mnb degeneration such that the size of the eye was indistinguishable from wildtype control. DYR533 was also found to suppress the rough eye phenotype of Tau (Fig. 2J) and Aβ42 (Fig. 2K) but not mnb (Fig. 2L) overexpressing eyes causing a significant increase in their size (Fig. 2M).

### DYRK1A inhibitors suppress the shortened lifespan caused by panneuronal overexpression of human Tau, human amyloid-β and fly DYRK1A (mnb)

**Figure 3:**
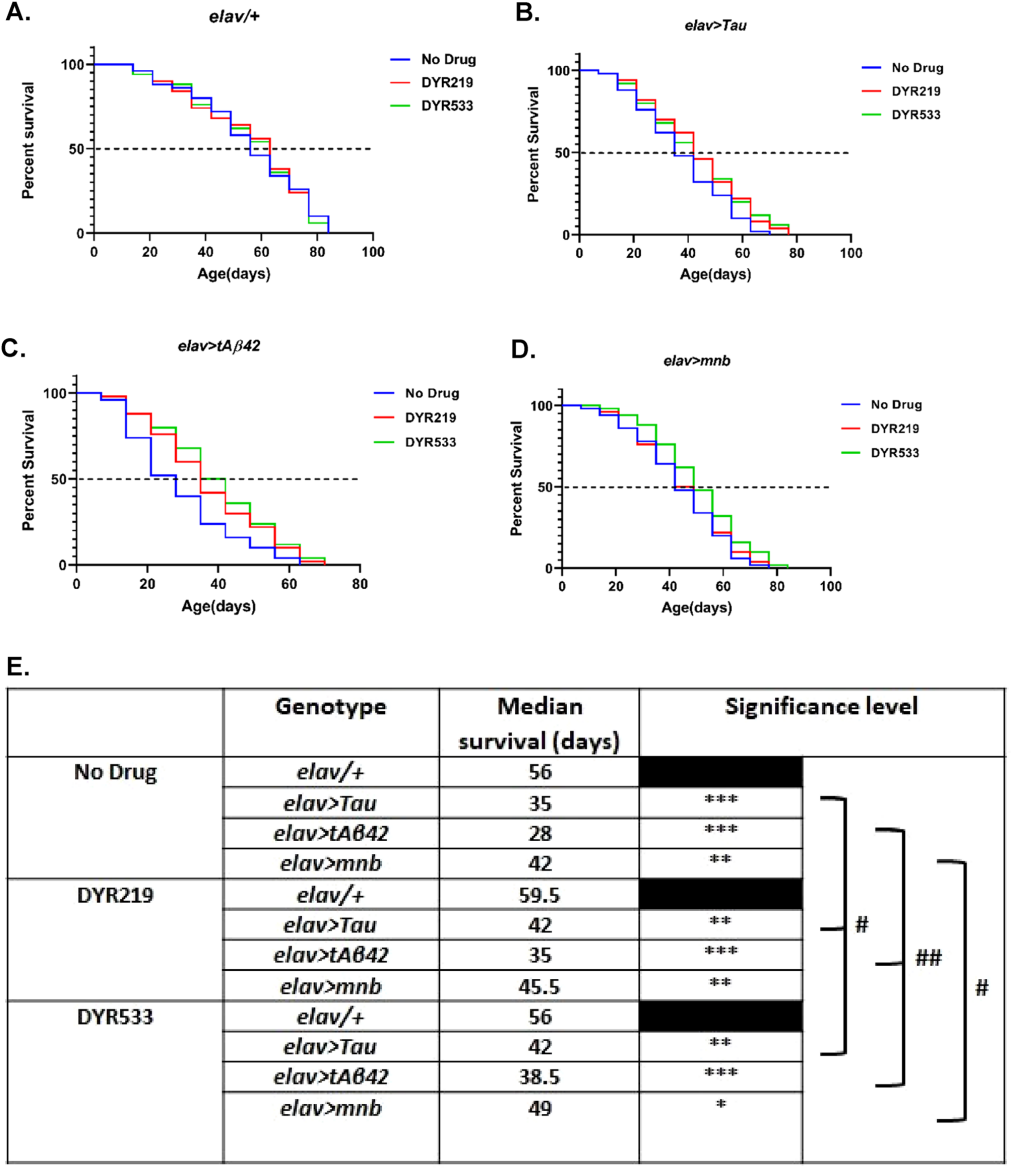
Pan-neuronal overexpression of human Tau, amyloid-β42 and mnb shortened lifespan which was partially reversible by DYRK1A inhibitor. Mantel-Cox (Log-rank) survival plots of (**A**) control (*elav-Gal4/* +) compared to flies overexpressing: human Tau (**B**), human tAβ42 (**C**) and fly mnb (**D**) off drug (blue lines) compared to treated with DYR219 (red) and DYR533 (green), a rightward shift in the curve indicates an extension of life. All genotypes and treatments were performed in parallel. (**E**) Table showing quantification of lifespan by median survival (days) which is reduced by pan-neuronal overexpression of Tau, tAβ42 and mnb compared to *elav-Gal4/* + control. DYR533 was found to be effective at extending the lifespan of all disease models while DYR219 lengthened the life of human Tau and human tAβ42 overexpressing flies (^#^*P* < 0.05 and ^##^*P* < 0.01 with n = 5 biological replicates per condition of 10 flies per replicate for all genotypes).

As DYR219 and DYR533 showed ability to suppress human Tau, Aβ42 and mnb degeneration we wished to see if the drugs could also treat other deficits caused by these AD and AD-DS associated genes. We first looked at longevity and found neither drug affected the viability and longevity of control (*elav-Gal4/* +) flies (Fig. 3A,E). However, pan-neuronal (*elav-Gal4*) overexpression of human Tau (38% shorter life), Aβ42 (50%) and mnb (25%) reduced (Fig. 3E) lifespan. DYR219 treatment lengthened lifespan of human Tau (Fig. 3B) and Aβ42 (Fig. 3C) overexpressing flies by 20% and 25% respectively (Fig. 3E). While DYR533 treatment lengthened lifespan of human Tau (Fig. 3B), Aβ42 (Fig. 3C) and mnb (Fig. 3D) overexpressing flies by 20%, 38% and 17% respectively (Fig. 3E).

### DYRK1A inhibitors suppresses locomotor deficits caused by panneuronal overexpression of human Tau, human amyloid-β and fly DYRK1A (mnb)

**Figure 4:**
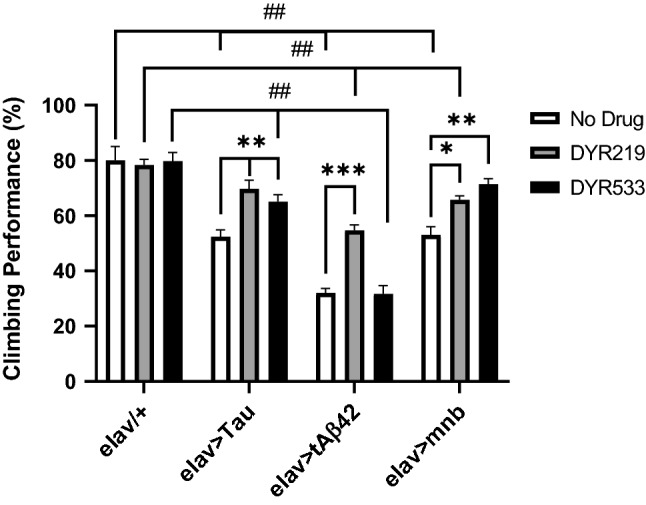
Pan-neuronal overexpression of human Tau, amyloid-β42 and mnb cause movement deficits suppressed by DYRK1A inhibitors. Without drug (white bars) pan-neuronal overexpression of human Tau 0N4R, tAβ42 and mnb decreased locomotion compared to control (*elav-Gal4/* +). Treatment with DYR219 DYRK1A inhibitor (grey bars) caused an increase in performance of all models, resulting in complete pharmacological rescue of human Tau overexpressing flies to control levels. DYR219 treatment significantly enhanced the climbing performance of all three degenerative mutants. Similarly, DYR533 treatment (black bars) significantly increased climbing performance of human Tau and mnb overexpressing flies fully rescuing mnb flies to control levels. 2-way ANOVA with Dunnett’s multiple comparisons with * and # as listed in the Methods statistics section and previous legends. n = 5 biological replicates per condition of 10 flies per replicate for all genotypes.

To confirm if our fly models had behavioural deficits related to the disease and whether the DYRK1A inhibitors might be effective at treating them, we tested locomotion using a negative geotaxis climbing assay^24,50^. A group of 2–5 days old control (*elav-Gal4/* +) flies are tapped to a bottom of a tube which elicits a reflex causing the flies to move away from gravity with about 75% of flies reaching the top of the tube within 10 secs (Fig. 4). Flies with pan-neuronal expression of neurotoxic human Tau, Aβ42 and mnb reduced climbing performance (Fig. 4). The DYRK1A inhibitor DYR219 did not affect normal behaviour of controls (*elav/* +) but did improve the locomotor deficits of all flies with pan neuronal overexpression of the different disease-associated genes, partially rescuing Aβ42 and mnb flies while fully rescuing Tau flies to a level indistinguishable from wildtype control (Fig. 4). DYR533 was also effective at suppressing the behavioural deficits of Tau and Aβ42 overexpressing flies, increasing their performance compared to the corresponding untreated genotype control but not to completely wildtype control levels.

### DYRK1A inhibitors suppress the sleep loss caused by clock neuron overexpression of human Tau, human amyloid-β and fly DYRK1A (mnb)

**Figure 5:**
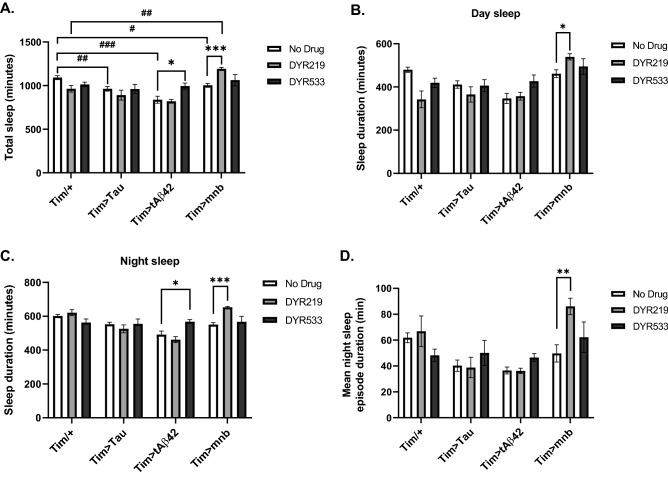
Overexpression of human Tau, tAβ42 and *mnb* throughout the clock caused sleep loss improved by DYRK1A inhibitors. (**A**) Clock-wide (*timeless (tim)-Gal4*) overexpression of human Tau, tAβ42 or mnb reduced total sleep compared to control (*tim/* +) off drug (white bars). DYR219 increased total sleep of mnb overexpressing flies and DYR533 increased total sleep of flies overexpressing tAβ42 in their clock. The total amount of sleep can be split into sleep occurring in the day (**B**) and night (**C**), overexpression of the disease associated genes reduced sleep both during the day and night. This revealed that DYR219 was effective at increasing sleep loss during the day and night of mnb overexpressing flies, while DYR533 treated the loss of nocturnal sleep of tAβ42. (**D**) Mean nocturnal sleep episode duration was measured and found to be increased by DYR219 treatment of flies with clock-wide overexpression of mnb. 2-way ANOVA with Bonferroni's multiple comparisons test (* P < 0.05, ** P < 0.01 and *** *P* < 0.001).

We then assessed sleep using *Drosophila* activity monitors (DAM) which count beam-crosses flies makes under different lighting conditions with sleep defined as more than five minutes inactivity in a 30 min time window with sleep mostly sleeping at night but also in the day when males often “siesta” during the afternoon^19,21^. Flies that overexpressed human Tau, Aβ42 and mnb throughout the clock (*Timeless (tim)-Gal4* promoter) decreased total (Fig. 5A), day (Fig. 5B) and night (Fig. 5C) sleep. DYR219 treatment increased sleep in mnb over-expressors, while DYR533 increased sleep of flies with clock Aβ42 overexpression (Fig. 5A). When sleep was split between that occurring during the day as opposed to at night it could be seen that DYR219 was able to increase sleep in the day (Fig. 5B) as well as night (Fig. 5C). While DYR533 increased nocturnal sleep of Aβ42 flies (Fig. 5C). Lastly DYR219 was also able to increase the duration of sleep episodes at night in mnb over-expressors (Fig. 5D). We found that the baseline level activity of the different genotypes on or off drugs were similar between groups, except for mnb overexpressors were less active on DYR219 (Supplementary Fig. S2), consistent with the increased sleep seen in these flies.

### DYRK1A inhibitors rescue memory loss caused by mushroom body overexpression of human Tau, human amyloid-β and fly DYRK1A (mnb)

**Figure 6:**
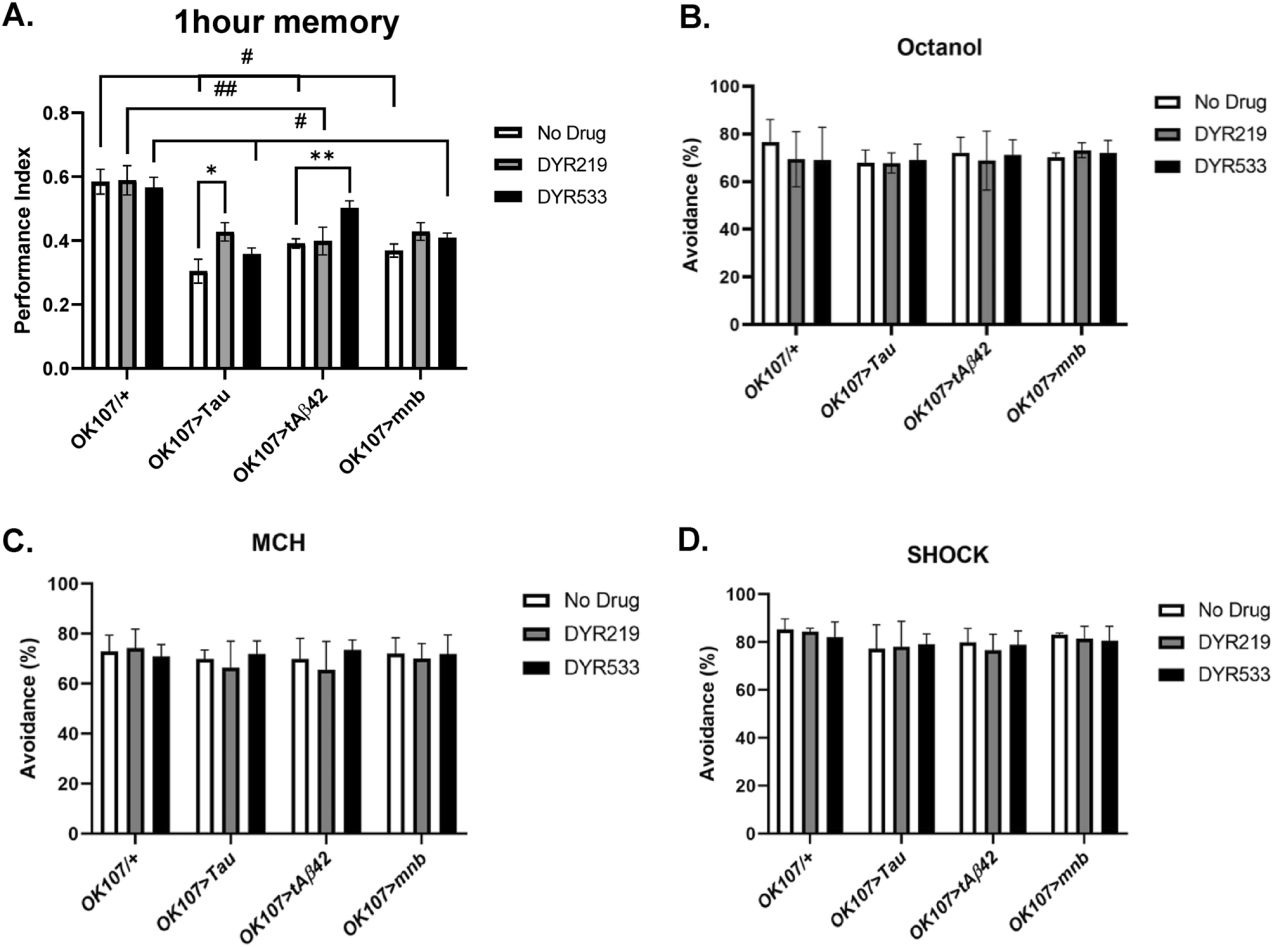
Mushroom body neuron overexpression of human Tau, tAβ42 and *mnb* cause memory deficits improved by DYRK1A inhibitors. (**A**) Overexpression of human tau, tAβ42 and mnb throughout the fly memory centre, the mushroom body (*OK107-Gal4*) decreased 1 h memory (performance index (PI)) compared to *OK107/* + control. DYR219 increased the memory performance of human Tau overexpressing flies and was able to completely rescue the memory loss of both human Tau and mnb overexpressors. DYR533 significantly increased the memory of human tAβ42 flies to a level indistinguishable than control. Data was analysed with 2-way ANOVA with Dunnett's post hoc multiple comparisons test, * and # as listed in the Methods statistics section and previous legends. Mean PI was taken from four independent experiments. Neither mushroom body overexpression of mnb, human tAβ42 and human Tau or DYR219 or DYR533 treatment change the response of the fly to (**B**) octanol, (**C**) MCH or (**D**) shock compared to control. Average % avoidance was taken from 3–4 independent experiments with each n = 30–50 flies per experiment per genotype.

To assess the hallmark loss of learning and memory of AD and DS we performed an olfactory-shock conditioning assay, where flies are exposed to two consecutive odours with which the first odour is paired with a mild foot shock and the second odour without. After an hour, the flies are taken to a choice point of a T-maze with one arm containing the odour previously paired with shock and the other the non-shocked odour, the flies display learning and memory by avoiding the shock-paired odour^23,24^. We found mushroom body memory neuron (*OK107-Gal4* promoter) overexpression of human Aβ42, Tau and mnb decreased 1 h memory (Fig. 6A). DYR219 treatment increased the memory of the tauopathic flies and DYR533 increased the memory performance of human Aβ42 flies, completely rescuing their memory to levels indistinguishable from wildtype (Fig. 6A). To check that the mushroom body overexpression of the genes and drug treatment did not disrupt sensory processes required to perform the task, we tested the ability of the flies to avoid the odours: octanol (Fig. 6B), MCH (Fig. 6C) and shock (Fig. 6D) used in the assay, all flies responded in manner indistinguishable from control, showing that the mutants had bona fide memory defects and that overexpression of the genes and drug treatments did not have non-specific effects in these assays.

## Discussion

We showed that neuronal overexpression of *Drosophila mnb,* the fly ortholog of DYRK1A had similar AD-DS relevant phenotypes as human Tau and Aβ42 including photoreceptor neuron degeneration, shortened lifespan, locomotor impairment, loss of sleep and memory loss. We found pharmacological blockade of the kinase activity of fly DYRK1A by DYR219 and DYR533 was effective at suppressing a range of these disease relevant phenotypes without having any adverse effect on control flies. This provides important phenotypic characterisation of mnb overexpression compared to human Aβ42 and Tau and the potential of DYRK1A inhibitors to treat such deficits.

We found DYR219 and DYR533 DYRK1A inhibitors suppressed degeneration caused by mnb, Tau and Aβ42 overexpression. We showed the DYRK1A inhibitors block mnb phosphorylation of human Tau at T231 and S396 for DYR219 and T231, S262 and S396 for DYR533. Thereby inhibiting the increased kinase activity caused by × 1.5 mnb overexpression but also the pathology caused by human Tau and Aβ42 we propose via decreasing endogenous mnb phosphorylation and pathological processing of these neurotoxic molecules. We used a very widely used *UAS-human MAPT (TAU 0N4R)* transgenic which was generated over 20 years ago^28^, however as mentioned earlier this *UAS-Tau 0N4R* line has been reported to be leaky i.e., shows expression independent of the Gal4 promoter^55^. We are satisfied that this is not a major concern because we have shown *UAS-Tau 0N4R/* + controls are wildtype for many of the phenotypes we report here^56^. Therefore, any potential Gal4 independent expression of the line is not sufficient to cause a phenotype relevant to this study.

We have also used the line multiple times and found it always gives a range of robust AD-related phenotypes^19,23,24,50,56^ and which are consistent with those reported here. We^19^ and others^25,57,58^ have also found the effects of *UAS-Tau 0N4R/* + are relatively consistent with other human *UAS-Tau* insertion lines (e.g. *UAS-Tau 2N4R*), again consistent with leakiness of this specific insert not being the main contributing factor to the phenotypes reported*.*

Confirming previous studies^24,28,50^ we found overexpression of AD-associated human Tau, Aβ42 and mnb (in this order of neurotoxicity) caused a rough eye phenotype, loss of photoreceptor neurons and a concomitant decrease in the size of the eye. This extends previous reports of that *DYRK1A* causes neurodegeneration in mice^36,37,39,41^ which then leads to loss of locomotor and cognitive performance in AD and DS which culminates in shortened life that is directly correlated with this pathology^30,33–35,41,59^. Interestingly in contrast to DYR219, DYR533 appeared to be able to suppress human Tau and Aβ42 degeneration independent of mnb, suggesting it may be able to directly block pathology via another mechanism. DYR219 was particularly effective at suppressing mnb mediated degeneration, completely rescuing eye size to that of wildtype control caused by mnb overexpression. This provides pharmacological validation of DYRK1A as a DS and AD therapeutic in a range of preclinical animal models. We do not know the reason for the differences in phosphorylation and phenotypic suppressive effects of DYR219 and DYR533. It might be that subtle differences in their chemistry, differences in their affinities, the concentration used and differences in circulating doses of the drugs in the fly, with DYRK1A being dose dependent in mice and human, with too little or much activity causing pathology^53,54^. For instance the drugs appear to have differential affects at inhibiting phosphorylation at S262, which is close to the first microtubule binding site so can modulate microtubule binding affinity, therefore phosphorylation at this site may be particularly associated with pathology^13^. In addition, the drugs may have differing effects on the different phenotypes studied which resulted from promoter misexpression of the different genes in different neuronal populations which may result in different levels of pathological gene products, differences in selective vulnerability of the different types of neurons and access by the drugs to the neurons.

We showed that lifelong exposure to the DYRK1A inhibitors had no detectable effect on lifespan of control, however they were found to be effective at suppressing the shortening of life caused by pan-neuronal overexpression of AD-associated Tau and Aβ42. We also found that although both drugs were able to suppress the human Tau and Aβ42, DYR533 as opposed to DYR219 could suppress shortened lifespan caused by mnb overexpression. Regardless DYRK1A inhibitors generally seem effective at suppressing the increased human Tau, Aβ42 and mnb senescence.

We also demonstrated that the neurotoxic effect of overexpression of Tau, Aβ42 and mnb had severe behavioural and cognitive consequences reversible by the drugs. We found locomotor performance was compromised in all the disease models, with the DYRK1A inhibitors suppressing motor deficits of all genotypes, except DYR533 was not able to reverse the effect of Aβ42. This may be due to differences in circulating dose and affinity of DYR533 which maybe insufficient to rescue Aβ42 mediated behavioural phenotypes. Alternatively perhaps the phenotypes cannot be completely explained by the degeneration of the neurons the neurotoxic genes are expressed in, for instance Aβ42 can also disrupt neuronal function in more subtle ways, such as disrupting neuronal excitability and Ca^2+^ handling^2,20,27,60^, which maybe differentially effected by the DYRK1A inhibitors. Regardless the DYRK1A inhibitors were partially effective at treating the motor deficits of the disease models and DYR533 was even able to fully rescue the motor performance of pan-neuronal mnb overexpressing flies to a level indistinguishable to control.

Clock-wide overexpression of Tau, Aβ42 and mnb reduced sleep both in the day and night with DYR219 increasing the sleep of mnb overexpressors in a level indistinguishable from wildtype, whilst DYR533 increased the sleep of flies overexpressing Aβ42 sleep. Intriguingly when the total sleep was split into day and night sleep, DYR219 was shown to increase day and night sleep in mnb overexpressor flies, while DYR533 fully corrected the loss of nocturnal sleep in Aβ42. Furthermore, DYR219 was able to make mnb overexpressor flies able to sleep for longer episodes at night, consistent with the drug improving consolidation and alleviating sleep fragmentation. It should be noted that people with AD suffer from sleep fragmentation as well as reporting difficulty sleeping at night making them susceptible to nocturnal wandering^4,5^. Our work complements previous work on the effect of clock expression of Tau and Aβ42 on circadian rhythms and sleep disruption in flies including Aβ42 and Tau sleep less during the day and night, while expression of tandem Aβ42 and Tau caused behavioural arrhythmia and sleep fragmentation^19–21,27,61,62^, and extends this to show that clock expression of mnb also causes sleep loss.

Our work is consistent with what is known in AD, which shows compromised clock function with post-mortem AD brain slices revealing extensive neurodegeneration of the mammalian clock, the suprachiasmatic nucleus (SCN) in human^8^ and rodent models of AD^63,64^. Prior to neurodegeneration flies and mice overexpressing human 0N4R tau neuronal hyperexcitability and inability to sleep at night^19,27,63,64^. Sleep has also been shown to remove Aβ42 and tau from interstitial fluid in rodents and cerebrospinal fluid in humans with levels increasing with wake time and being removed at night with sleep deprivation further exasperating pathological leading to tau spreading^8,65^. Therefore, DYRK1A inhibitors have potential as correctors of DS and AD associated sleep problems thereby causing a predicted increase in Aβ42 and Tau clearance, slow pathology and thereby boast cognition^8,65^. This work also validates the use of *Drosophila* as a high throughput system to screen for novel DS and AD circadian and sleep drugs^61,66^. Therefore, DYRK1A inhibitors have potential to be tested as sleep correctors for DS that would likely also have beneficial effects on cognition and motor skills^6^. Likewise, the drugs may also be effective for sleep disruption in AD with the additional potential benefit of correcting sun-downing, when the patients’ symptoms, anxiety and confusion are greater in the evening. Similarly, the inhibitors may suppress AD nocturnal insomnia, the increase in nocturnal wandering and associated poorer cognitive measures the next day^67–69^.

It is known that robust sleep–wake cycles improve memory function and sleep is required for long term memory consolidation^70^. Because of this and the fact that learning and memory difficulties are hallmarks of DS and AD we tested the effect of mushroom body (the memory centre of the fly) overexpression of Tau, Aβ42 and mnb which reduced memory. DYR219 suppressed the memory loss caused by human Tau completely rescuing performance to wildtype levels while DYR533 suppressed human Aβ42 memory loss completely rescuing it to control levels. DYR219 also rescued fly mnb mediated memory loss. This suggests that DYRK1A inhibitors have potential as use as cognitive enhancers for DS and AD. We also performed sensory controls that showed that the drugs had no adverse effects on olfaction or response to reinforcement.

Again, we found the two DYRK1A inhibitors herein showed differential benefits on the different phenotypes of the models tested, likely due to their differences in inhibition of mnb mediated phosphorylation and because the different transgenes could have different neurotoxic effects in the different types of neurons that they were expressed in e.g. eyes (*GMR-Gal4*) compared to all neurons (*elav-Gal4*), clock neurons (*tim-Gal4*) and mushroom body memory neurons (*OK107-Gal4*). Again, suggesting that different types of neurons may display selective vulnerability to the neurotoxic effects of the different disease associated gene, with expression of human AD genes being more detrimental than expression of fly mnb. Therefore, the more extreme phenotypes of the human AD genes might be more difficult to fully rescue with a DYRK1A inhibitor, than the more subtle defects caused by fly mnb overexpression, for which the drug was designed. Likewise, this reiterates that the drugs may have different effects or access to the different neurons the genes are expressed in for instance, the photoreceptor neurons are peripheral and sensory, while the later promoter lines express in possibly less drug accessible central neurons. Future work could be aimed at optimising the dose and timing and treatment for the different models and assays.

Our findings show mnb is functionally conserved with DYRK1A, with mice overexpressing mouse *Dyrk1a* or human *DYRK1A* having similar motor and cognitive deficits, suggesting that triplication of *DYRK1A* likely contributes to pathology and behavioural deficits in DS and AD-DS^36,37,39,41^. Overexpression of these disease associated genes not only cause neurodegeneration but also may disrupt neuronal excitability and synaptic plasticity^19,23,24,50^, contributing to the loss of sleep and memory seen. This reiterates that molecular mechanisms underlying these behaviours are well conserved between flies and mammals including humans, allowing the genetic tractability, high throughput assays, and rapid ageing of *Drosophila* to study these processes and screen for new drugs for these poorly treated diseases reducing the use of large numbers of mammals for such screens.

## Methods

### Fly stocks and husbandry

Flies were raised on a standard corn yeast cornmeal diet (0.7% agar, 1.0% soya flour, 8.0% polenta/maize, 1.8% yeast, 8.0% malt extract, 4.0% molasses, 0.8% propionic acid and 2.3% nipagen) at 25 °C on 12 h light:12 h dark (LD) cycles and. *CSw*^*-*^ was used as wild type control flies a kind gift of Dr Scott Waddell (University of Oxford, UK). *Tim(27)-GAL4/CyO*^19^ was a generous gift of Dr Ralf Stanewsky (University of Münster, Germany). The following strains were obtained from Bloomington *Drosophila* Stock Center (BDSC; stock number provided in brackets): *elav-Gal4/CyO* (8765) *OK107-Gal4* (854) and *GMR-Gal4/CyO* (9146). *UAS-human MAPT (TAU 0N4R) wild-type* was kindly given by Dr Linda Partridge, University College London)^28^, *UAS-human tandem Aβ42-22 amino acid linker-Aβ42*^21^ (gift from Dr Damian Crowther, University of Cambridge) and *UAS-mnb* flies (*minibrain-H*, CG42273^48^) were kindly provided by Dr Kweon Yu (Korea Research Institute of Bioscience and Biotechnology). Fly strains including *Tim-Gal4, OK107-Gal4, elav-Gal4* and *UAS-Tau* that were routinely used for behaviours that might be affected by genetic background, were fully cantonised for this study, as described previously^56^, a study where we found cantonisation had little effect^56^ on many of the phenotypes reported here.

### Pharmacology

The small molecule DYRK1A inhibitors, DYR219 (with DYRK1A dissociation constant (K_D_) of 16 nM) and DYR533 (K_D_ = 1.4 nM) were prepared in multi-gram quantities^53,54^ and are available on request from Dr Christopher Hulme (hulme@pharmacy.arizona.edu) under MTA with University of Arizona. Dissociation constants were determined using the DiscoveRx K_D_ Elect assay described at www.discoverx.com. Both compounds were evaluated for selectivity against 468 kinases via KinomeScan™ technology at 1 μM (DYR219 S(35)-selectivity was 0.19 compared to DYR533 S(35)-selectivity being 0.032). Based on the concentrations of DYR219 (and similar DYRK1A inhibitors) in food that were found to be effective at suppressing neurodegeneration and cognitive deficits of 3xTg-AD and other mice^45,46,51^ we dissolved 100 mg of DYR219 and DYR533 in aqueous solution and then mixed the drug solutions into cooling (~ 40 °C) liquid fly food producing a final concentration of 304 μM (DYR219) and 248 μM (DYR533). Flies laid onto the drug food with the offspring developing and feeding on the drug throughout their lives including during phenotypic testing.

### Western blotting

Whole fly heads of mixed sex flies were dissected and placed in RIPA Lysis Buffer (Thermo Scientific) with 1:100 Protease/Phosphatase Inhibitor Cocktail (Cell Signalling) and homogenized. After 15 min 12,000 rpm centrifugation at 4 °C, the supernatants were collected, and protein concentrations determined using a NanoDrop spectrophotometer (Thermo Fisher Scientific).

Protein samples were then prepared by adding 1:4 of 4 × Bolt™ LDS Sample Buffer (Thermo Scientific) and 1:10 of 10 × Bolt™ Sample Reducing Agent (Invitrogen) followed by heating at 95 °C for 3 min. Samples of 100 µg total protein were separated by sodium dodecyl sulfate–polyacrylamide gel electrophoresis using precast 4%-12% Bolt Mini gels were transferred to PVDF membrane (Thermom Scientific). The membranes were blocked overnight at 4 °C in blocking solution (Tris-buffered saline [TBS]: 20 mmol/L Tris (pH 7.4), 150 mM NaCl, with 0.1% Tween 20 [TBS with Tween (TBST)] and 5% [wt/vol] bovine serum albumin (BSA)) and incubated at 4 °C overnight with the primary antibody: mouse anti-Tau (#T9450, Sigma), mouse anti-β-actin (#A2228, Sigma-Aldrich, 1:1000), anti-Tau phospho- S262, S356, S396 or T231 (Abcam) (1:500) in TBST containing 1% BSA. After three washes with TBST, the blots were incubated for 1 h at room temperature with horseradish peroxidase–conjugated mouse or rabbit IgG secondary antibody (1:2000, Cell Signalling) and then washed three times with TBST. Detection was performed using Western ECL Substrate (GE) according to the manufacturer's instructions and developed on X-ray films first and then scanned. The relative protein expression levels were quantified by densitometry using ImageJ Gel Analysis software. Western blots from at least three independent biological replicate experiments for each fly strain were used for quantification.

### Eye degeneration assay

Overexpression of transgenes in the developing and adult photoreceptor neurons was driven by the *Glass multimer reporter* (*GMR-Gal4*) promoter line. 2–5 day old female flies were CO_2_ anesthetised and ethanol immersed to euthanise the fly preventing movement during image capture^71^. A Zeiss SteREO Discovery.V8 stereomicroscope (with up to 8 × magnification) attached to a Zeiss AxioCam MRm camera was used to image and assess the qualitative degenerative phenotype of “rough eyes” and quantify surface area using Zeiss Zen software which was analysed with 2-way ANOVA with multiple comparisons tests.

### Survival assay

Five groups of 10 two days old-mated females (who had spent the previous day with males) were housed in vials containing standard food with or without drug at 25 °C, with all the genotypes and treatments being performed in parallel. The sex and mating status of the fly affects lifespan therefore we standardize across groups by picking mated females. The number of deaths was counted appropriately every two days with the remaining flies transferred to a new food vial with or without drugs^55^. Mantel-Cox survival curves were plotted and log-rank tests performed to compare survival between genotypes and drug treatment groups.

### Climbing assay

Five groups of 10 two-five days old male flies were collected for each genotype and drug treatment group. Although the sex of the fly does not influence climbing, to save animals we use the males from the progeny of the *elav* crosses used for the survival assay. They were given ~ 1 h to acclimatise to a standard empty food vial at 25 °C. Flies were gently tapped to the bottom of the 7.5 cm plastic vial this initiated the negative geotaxis reflex allowing the number of flies that crossed a line drawn 2 cm from the top of the tube in 10 s to be counted. This was then expressed as a % referred to as the climbing performance with 2-way ANOVA with Dunnett’s multiple comparisons being employed to analyse data^24,50^.

### *Drosophila* activity monitoring for sleep

Two-five days old male flies were put into single tubes in *Drosophila* Activity Monitoring (DAM) system (DAM2, TriKinetics Inc, USA) this allowed quantification of sleep based on activity data from five days of 12 h:12 h LD which is counted in one and 30 min bins. The sex of the fly does affect sleep, and we pick males as they do not produce progeny whose activity would confound activity monitoring. Sleep was defined as bouts of inactivity lasting more than five minutes as per convention^72^. The mean total sleep, mean sleep in the day and night and sleep episode length were calculated for each individual using the Sleep and Circadian Analysis MATLAB Program (SCAMP) in MATLAB^19^.

### Olfactory shock conditioning

20–40 (mixed sex, as the sex of the fly does not affect memory) two-five days old flies were collected and acclimitised to LD 25 ºC and 70% relative humidity conditions. The olfactory shock conditioning assay was performed under dim red light, to which the flies cannot see allowing them to concentrate on odour cues. Flies were placed in the training tube lined with an electrifiable grid and given 90 s rest in a stream of fresh air, prior to exposure to the first dour (conditioned stimulus, CS^+^) which was paired with twelve 70 V DC electric shocks (unconditioned stimulus, US) for one minute. The flies were then given 45 s rest of fresh air to clear the tube of CS^+^ before being exposed to the second odour (CS^-^) without being given any electric shock. The odorants used were either 3-octanol (OCT, Sigma) or 4-methylcyclohexanol (MCH, Sigma) diluted in 10 ml mineral oil and adjusted to a concentration (~ 1:1000) which was equally aversive to the flies. After a 1 h rest on food intermediate-term memory (ITM) was measures using a performance index (PI) which was calculated as follows:

$$PI= \frac{({N}_{CS-}-{N}_{CS+})}{({N}_{CS-}+{N}_{CS+})}$$

where N_CS−_ and N_CS+_ is the number of flies choosing CS^−^ and CS^+^ arms of a T maze. The CS^+^ odour was reversed in alternate groups of flies to minimise odour odorant with the mean of the performance between the two consecutive odour trials giving a n = 1 (i.e., 40–80 flies). The olfactory and shock reactivity of the different genotypes on and off drug were performed as sensory controls. For shock reactivity, flies were placed at the choice point of a T maze of two shock tubes, one of which delivered the shock protocol above. The number of flies avoiding the shock over the total number of flies in the trial was used to generate a % shock avoidance. Likewise, % avoidance of the concentration of the respective odours versus air was calculated.

### Statistical analysis

Data were analysed using GraphPad Prism (version 8.00 for Windows, GraphPad Software, La Jolla California USA) with normality checked for all datasets using the Shapiro–Wilk’s test, prior to selection of the appropriate parametric or non-parametric statistical test. The tests used and the number of animals (n) used for each dataset is given in the corresponding figure legend. Data is presented as Mean ± Standard error of the Mean (SEM) and statistical levels shown as following non-significant (ns) *P* > 0.05, * or ^#^*P* < 0.05, ** or ^##^*P* < 0.01 and *** or ^###^*P* < 0.001.

## Supplementary Information

Supplementary Figures.

## Data Availability

The datasets generated and/or analysed during the current study are available in the data.bris.ac.uk/data repository using the https://doi.org/10.5523/bris.2b6t0stfalwkz2bmzj29f0jqsp.
