# Supplementary material for: DYRK1A antagonists rescue degeneration and behavioural deficits of in vivo models based on amyloid-β, Tau and DYRK1A neurotoxicity
Source: Sci Rep. 2022 Sep 23;12:15847. doi: 10.1038/s41598-022-19967-y (PMC9508268; doi:10.1038/s41598-022-19967-y)
Supplement: Supplementary file 1 — Supplementary Figures. [file 41598_2022_19967_MOESM1_ESM.docx]

**Supplementary Figure 1.**

**A.**


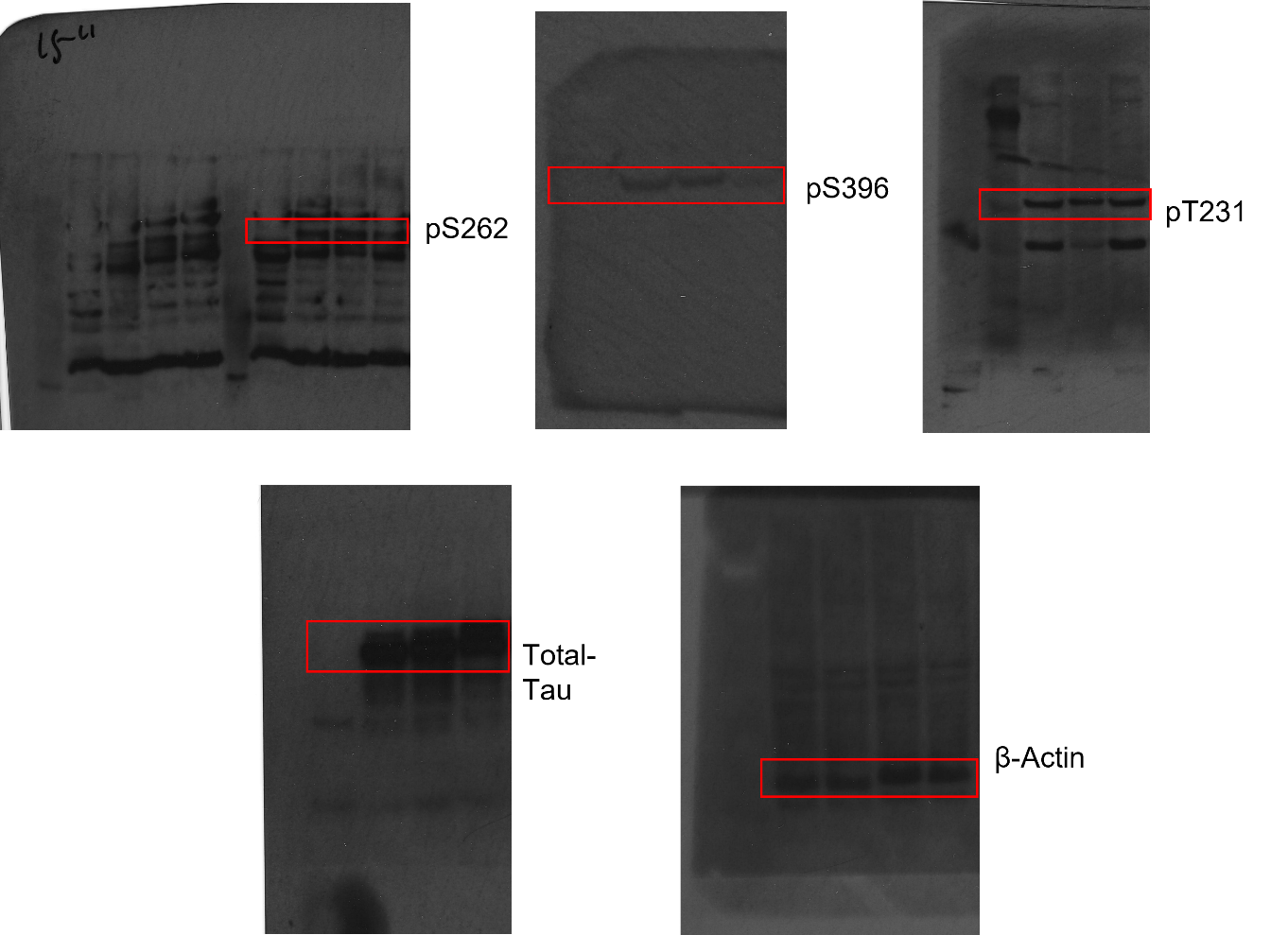


**C.**

**B.**

**E.**

**D.**

**Supplementary Figure 1. DYRK1A inhibitors reduced phosphorylation of human Tau expressed pan-neuronally in *Drosophila***

Whole Westerns for images (red boxes) used in Figure 1. Western blots show effects of treatment with DYRK1A inhibitors (DYR219 and DYR533) on phosphorylation of human Tau 0N4R overexpressed pan-neuronally using the *elav-Gal4* driver: 1^st^ lane is *elav-Gal4/+* control, 2^nd^ lane is *elav>human Tau* *0N4R* with normal food, 3^rd^ lane is *elav>human Tau* treated with 304 μM DYR219 and 4^th^ lane is *elav>human Tau* with 248 μM DYR533 treatment throughout development and adulthood (treatments were the same for all figures). Three antibodies against phosphorylated Tau (pS262, pS396 and pT231) were tested compared to the level of total unphosphorylated Tau (~55kDa) using a fourth antibody.

**Supplementary Figure 2.**

**A.**

**B.**

**C.**

**Supplementary Figure 2. Quantification of activity of *Drosophila* with clock expression of human *Tau, tAβ42* and *mnb***

Flies with clock-wide (*timeless (tim)-Gal4*) expression of human Tau, tAβ42 or mnb were placed in *Drosophila* Activity Monitors (DAM) under LD conditions allowing total activity counts to made throughout a 24hr day (A) and then plotted as activity during the day (B) and night (C). This showed and DYR219 significantly decreased total activity counts of mnb overexpressing flies under all three conditions, this was consistent with the increased sleep seen in mnb flies treated with DYR219. One-way ANOVA with Bonferroni's multiple comparisons test (*** P* < 0.01 and *** *P* < 0.001).
